# LONG-TERM CARE CONCERNS AMONG CHILDLESS OR SINGLE CAREGIVERS TO PEOPLE LIVING WITH DEMENTIA

**DOI:** 10.1093/geroni/igad104.2851

**Published:** 2023-12-21

**Authors:** Elyse Couch

**Affiliations:** Brown University, Providence, Rhode Island, United States

## Abstract

It is estimated that 77% of people living with dementia receive help with self-care and household activities from informal caregivers (typically family members), meaning there is a significant proportion of the population not receiving informal support. In this qualitative study, we aimed to explore similarities and differences in family caregivers’ concerns about organizing their own care should they develop dementia in the future between those who were childless and/or single and those who had a current partner and children. We interviewed 12 British current and former dementia family caregivers, 5 of whom were childless and/or single, 9 were women and 3 were men, with a mean age of 61. All 5 childless and/or single participants were women. Participants perceived a caregiver as vital for ensuring the person with dementia received the care they needed from services. Caregivers who were childless and/or single expressed concern that by not having someone to be their caregiver should they develop dementia in the future, they would be vulnerable to receiving poor care. In these cases, childless and/or single caregivers described the possibility of exploring options for suicide or euthanasia should they be diagnosed with dementia in the future. Similar sentiments were not expressed by caregivers with current partners and children. While this study uses a small sample size, these findings highlight persons without children and/or a partner have serious concerns about their future care, should they develop dementia. More research is needed to understand how to better support people with dementia without caregivers.

